# Initial Sublethal Exposure to an Argentine *Bacillus thuringiensis* Strain Induces Chronic Toxicity and Delayed Mortality in *Alphitobius diaperinus* (Coleoptera: Tenebrionidae)

**DOI:** 10.3390/insects17020213

**Published:** 2026-02-18

**Authors:** Gisele Ivonne Antonuccio, Lucas Candás, Diego Herman Sauka

**Affiliations:** 1 Instituto Nacional de Tecnología Agropecuaria (INTA), Instituto de Microbiología y Zoología Agrícola (IMYZA), Buenos Aires B1686IGC, Argentina; candas.lucas@inta.gob.ar (L.C.); sauka.diego@inta.gob.ar (D.H.S.); 2 Servicio Nacional de Sanidad y Calidad Agroalimentaria (SENASA), Buenos Aires C1107ADR, Argentina; 3 Consejo Nacional de Investigaciones Científicas y Técnicas (CONICET), Buenos Aires C1425FQB, Argentina

**Keywords:** *Alphitobius diaperinus*, *Bacillus thuringiensis*, sublethal effects, biocontrol, virulence

## Abstract

*Bacillus thuringiensis* is the most extensively studied entomopathogenic bacterium worldwide; however, its sublethal effects on beetles remain poorly characterized. The aim of this study was to evaluate the toxicity of a previously selected Argentine strain of *B. thuringiensis* on second-instar *Alphitobius diaperinus* larvae during an initial 14 days of exposure, and to assess its effects at day 14 and throughout the remainder of the life cycle until death. Three treatments were applied: control, LC_30_, and LC_50_. Larval, pupal, and adult weight and body surface area were recorded, and nutritional composition was quantified using colorimetric methods. Insect status was monitored every 48–72 h over a total period of 540 days, until the death of the last individual. Among the evaluated variables, statistically significant differences between control and treatment groups were detected in larval area and weight, in the survival analysis and in two nutritional components: total protein and lipid content per larva. Overall, the results demonstrate that initial sublethal exposure to *B. thuringiensis* induces chronic lethal effects with delayed mortality in *A. diaperinus*, indicating irreversible physiological damage. This provides valuable information not only for understanding the biology of this insect but also for stakeholders involved in the productive scaling of beetle-targeted bioinputs.

## Simple Summary

Pest control in agriculture and livestock is a constant challenge, particularly when insect pests affect animal production systems. Although agrochemicals have traditionally been the main control strategy, environmentally friendly alternatives are increasingly needed. *Bacillus thuringiensis* is a widely used bacterium for insect control that acts when ingested and is valued for its safety and target specificity. However, the initial effects of concentrations that do not immediately kill insects but weaken them over time have been little studied in beetle pests. In this work, we evaluated the initial sublethal effects of an Argentine *B. thuringiensis* strain on *Alphitobius diaperinus* larvae after 14 days of dietary exposure and followed the insects throughout their life cycle to assess chronic toxicity. Larvae exposed to the bacterium showed significant reductions in weight and body size, altered nutritional reserves, and reduced survival compared with untreated individuals. Even insects that initially survived exhibited significant delayed mortality, indicating long-term irreversible damage. These results demonstrate that *B. thuringiensis* can reduce beetle populations not only by killing insects directly but also by weakening them through chronic effects, supporting its use as an effective and sustainable biotechnological tool for pest management.

## 1. Introduction

The lesser mealworm, *Alphitobius diaperinus* Panzer (Coleoptera: Tenebrionidae) [1], is a key pest in poultry production systems. In addition to causing direct damage to facilities, it is recognized as a potential vector of avian pathogens, including bacteria, viruses, and parasites, posing significant sanitary risks and potentially leading to substantial economic losses [2]. The intensive use of chemical insecticides for its control has resulted in resistant populations, as well as concerns about residues in poultry products and risks to animal and human health. These challenges underscore the need for alternative, environmentally safe control strategies [3,4].

Among microbial control agents, *Bacillus thuringiensis* is the most widely used entomopathogenic bacterium. During sporulation, it produces parasporal crystalline inclusions composed of proteins (Cry and Cyt) that exhibit selective toxicity against insect larvae upon ingestion. This bacterium can also secrete pesticidal proteins during the vegetative stage (Vpa/Vpb and Vip). To date, hundreds of *B. thuringiensis* pesticidal proteins have been described [5], some of which have been developed into bioinsecticide formulations or expressed in transgenic crops. Their activity spans multiple insect orders, including Lepidoptera, Diptera, Coleoptera, and Hemiptera.

The first *B. thuringiensis* strain with activity against coleopterans was reported by Krieg et al., 1983 [6]. Since then, several pesticidal proteins have been associated with toxicity to *A. diaperinus* larvae [7,8]. Nevertheless, compared with lepidopteran pests, the market for coleopteran-targeting bioinsecticides remains less developed, partly due to the historical focus on caterpillars, the limited availability and narrower activity of coleopteran-active *B. thuringiensis* toxins, and the cryptic feeding habits of many beetle larvae, which reduce their exposure to *B. thuringiensis* and complicate effective application. Recent evaluations of local *B. thuringiensis* strains have identified promising candidates for controlling *A. diaperinus*. Pérez et al., 2025 [9] selected INTA Mo4-4 as highly toxic to larvae, demonstrating that its insecticidal activity is predominantly associated with the spore–crystal pellet, consistent with the involvement of Cry proteins. In that study, INTA Mo4-4 showed the highest toxicity among 41 evaluated strains and caused mortality levels 2.7-fold higher than those of the reference strain *B. thuringiensis* svar. *morrisoni* tenebrionis DSM 2803. Previous reports have shown that such parasporal crystal proteins—including Cry3Aa, Cry3Bb, Cry8Ca and proteins with dual activity against Diptera and some coleopterans, including Cry4B, Cry10, Cry11A and Cyt1A—constitute the main virulence factors of *B. thuringiensis* against *A. diaperinus* [7,8].

However, most studies have focused on acute toxicity, while the potential sublethal effects of *B. thuringiensis* exposure remain largely unexplored [10,11,12,13]. This is particularly relevant because, under field or farm conditions, environmental factors such as UV light, rainfall and microbial degradation often reduce the persistence and availability of *B. thuringiensis* toxins [14]. As a result, insects may be exposed to initial sublethal doses that, although insufficient to cause immediate mortality during the early stages of exposure, could affect development, reproduction, and overall fitness. To date, studies addressing such sublethal effects in *A. diaperinus* are scarce. Understanding how initial sublethal concentrations of *B. thuringiensis* impact this pest could provide valuable insights for integrated pest management and contribute to more sustainable control strategies. Therefore, the objective of this work was to evaluate the initial sublethal effects and subsequent chronic toxicity of an Argentine *B. thuringiensis* strain on the development, survival and fitness of *A. diaperinus*.

## 2. Materials and Methods

### 2.1. Production of Bacillus thuringiensis INTA Mo4-4 Active Ingredient

INTA Mo4-4 is an Argentine *B. thuringiensis* strain isolated from stored-product dust collected in the locality of Chacabuco, Buenos Aires Province, Argentina. The strain is preserved in the Bacterial Collection of the Instituto de Microbiología y Zoología Agrícola, Instituto Nacional de Tecnología Agropecuaria (IMYZA-INTA), as previously reported by Pérez et al. (2025) [9]. *Bacillus thuringiensis* INTA Mo4-4 biomass was produced following the protocol of Pérez (2017) [15] with minor modifications. An optimized BM broth (containing 2.5 g NaCl, 1 g KH_2_PO_4_, 2.5 g K_2_HPO_4_, 0.25 g MgSO_4_·7H_2_O, 0.1 g MnSO_4_·H_2_O, 5 g glucose and 6 g yeast extract per liter of distilled water, adjusted to pH 7.2) was prepared and divided into 12 Erlenmeyer flasks (50 mL per flask). Each flask was inoculated with 50 μL of a highly concentrated stock suspension of the spore-crystal complex. Cultures were incubated in the dark at 28 °C with shaking (250 rpm) for 72 h, until autolysis occurred. The biomass (spore-crystal complex) was collected by centrifugation (10,000 *g*, 4 °C, 20 min), washed three times with sterile distilled water, dried at 28 °C for four days, and ground to a fine powder that was stored at −20 °C until further use in subsequent analyses and bioassays.

### 2.2. Bioassays for Toxicity

Biological tests with a spore-crystal complex suspension were conducted, except that a series of six concentrations (concentration range: 37.13–320 μg/mL; dilution factor: 0.65) were prepared to establish the concentration-response relationship by Probit analysis. Forty-eight larvae (24 larvae per plate) were tested for each concentration and bioassay date.

Bioassays were performed against second instar larvae of *A. diaperinus* using the diet incorporation method previously described Pérez (2017) [15]. Artificial larval diet was prepared daily (133.3 g chicken feed, 10 g agar, 1 L deionized water) and sterilized (121 °C, 15 min). Preservatives (ascorbic acid 2.5 g/L, sorbic acid 1.25 g/L, nipagin 2.08 g/L) were added after cooling to 55 °C. Strain suspensions were incorporated into freshly prepared diet made on the same day, based on chick starter feed (4 mL per 36 mL diet per Falcon tube), and 400 μL of diet were dispensed per well in 24-well plates. Second-instar larvae were individually placed in wells. Mortality was recorded after 14 days at 29 °C. Four independent bioassays fulfilling the statistical criteria for *B. thuringiensis* were chosen as described by Iriarte & Caballero 2001 [16], and LC_30_ and LC_50_ values were estimated using Probit analysis [17] in IBM SPSS Statistics v19. To ensure robustness and reproducibility, only bioassays showing coefficients of variation ≤ 20% were considered valid.

Two series of bioassays were performed: first, six concentrations of spores and crystals were tested for acute toxicity; second, sublethal bioassays using LC_30_ and LC_50_ concentrations were conducted on surviving larvae to assess effects on development and fitness.

The selection of LC_30_ and LC_50_ was based on bibliographic references that revealed sublethal effects of *B. thuringiensis* on pest insect larvae, both individually and in combination with other control strategies [18,19].

### 2.3. Evaluation of Sublethal and Chronic Effects

Initial sublethal effects were evaluated after 14 days of exposure to the wet diet (LC_30_ and LC_50_, as defined in Section 2.2). To quantify growth inhibition, larvae were weighed in pools of 48 individuals and photographed in groups of four to estimate their body area using Image J software (version 1.54g; National Institutes of Health, Bethesda, MD, USA). Following this initial exposure phase, individuals were transferred to and maintained in separate test tubes with a dry (untreated) diet to prevent cannibalism and ensure precise individual tracking, while allowing for the assessment of delayed mortality. *A. diaperinus* specimens were monitored throughout their entire life cycle. Survival, molting, pupation, and adult emergence were recorded every 48–72 h. Upon reaching these stages, pupae and adults were weighed and photographed. Sex determination at the pupal stage followed the morphological criteria of Esquivel et al., 2012 [20].

To identify chronic toxicity and determine the maximum life expectancy for *A. diaperinus* per treatment, monitoring was extended for up to 540 days from hatching. This period was established based on the maximum lifespan recorded in the laboratory conditions, where 0.42% of the control, 0.46% of the LC_30_, and 0.55% of the LC_50_ populations reached this age. This extended observation window is essential to capture physiological “hidden costs” and delayed mortality—which define here as chronic lethal effects—that standard short-term bioassays typically overlook, providing a comprehensive view of the long-term impact of *B. thuringiensis* exposure.

Survival analysis was conducted using Kaplan-Meier curves, and statistical differences among treatments were assessed using the log-rank (Mantel-Cox) test applying a Bonferroni correction for multiple comparisons. To evaluate the rate of mortality within each treatment, lethal time (LT_50_ and LT_90_) values were estimated using bootstrap confidence intervals [21].

### 2.4. Biochemical Analyses of Surviving Larvae

Surviving larvae from control, LC_30_, and LC_50_ treatments were randomly pooled and sacrificed for biochemical assays to evaluate the impact of Bt exposure on energy reserves.

**Proteins:** Following the protocol of Brogdon 1984 [22], samples (pools of 2–14 larvae, depending on size) were homogenized in phosphate-buffered saline (PBS, pH 7.4) and centrifuged at 15,400× *g* for 3 min at 4 °C to separate the supernatant from the pellet. The resulting supernatants were analyzed using Bradford reagent in 96-well plates. Protein concentrations were calculated from bovine serum albumin (BSA) standard curves and normalized to larval biomass (µg protein/mg larval weight) by measuring absorbance at 595 nm using a microplate reader.

**Lipids:** Total lipids were extracted as described by Anschau et al., 2017 [23] with slight modifications. Pools of ~10 larvae were homogenized in chloroform:methanol (2:1, *v*/*v*) mixture. After centrifugation (15,400× *g* for 3 min at 4 °C) to separate the supernatant from the pellet, supernatants were reacted with concentrated H_2_SO_4_ and vanillin-phosphoric acid reagent for colorimetric quantification. Absorbance was recorded at 530 nm, and lipid content was expressed as µg/mg of larval weight.

**Sugars and glycogen:** According to Yuval et al., 1998 [24] sugars were extracted using a chloroform:methanol (1:2, *v*/*v*) solution, whereas glycogen was obtained by aqueous extraction from the resulting pellets in a subsequent step. Both analytes were reacted with anthrone in H_2_SO_4_ using different reagent proportions following the reference method, and absorbance was measured at the same wavelength (625 nm). Sugars were quantified using glucose-based standard curves, while glycogen standards (Fermentas, molecular biology grade, 20 mg mL^−1^) were used for glycogen determination.

For all variables analyzed in this study, including biological parameters and biochemical assays, statistical assumptions (normality via Shapiro-Wilk and homogeneity of variance via Levene’s test) were verified to ensure the appropriateness of the statistical tests. Differences among treatments were analyzed using one-way analysis of variance (ANOVA) for parametric data or the Kruskal–Wallis test for non-parametric data, with Bonferroni corrections applied where appropriate.

## 3. Results

### 3.1. Lethal and Sublethal Concentration Estimates

Concentration–mortality responses for the four bioassays are summarized in Table 1. LC_30_ values ranged from 61.55 to 84.34 µg/mL, and LC_50_ values ranged from 117.83 to 154.55 µg/mL. The slopes of the probit regressions varied between 1.42 and 2.18. χ^2^ values (4 df) indicated a satisfactory model fit across all assays. Coefficients of variation were below 20% for both calculated LC levels. The mean LC_30_ (68.91 µg/mL) and LC_50_ (135.74 µg/mL) were selected as the initial sublethal exposure levels for subsequent experiments.

### 3.2. Effects on Larval Performance and Development

The impact of initial sublethal concentrations (LC_30_ and LC_50_) of the spore-crystal suspension of INTA Mo4-4 on selected biological parameters of *A. diaperinus* is summarized in Table 2. Additional data are provided in the Supplementary Information, including Table S1 (Individual larval weight), Table S2 (Individual larval area), Table S3 (Individual larval and pupal stage duration), and Table S4 (Pupae and adult area and weight).

Sublethal exposure to *B. thuringiensis* INTA Mo4-4 at LC_30_ and LC_50_ produced clear effects on larval growth. Both concentrations significantly reduced mean larval weight and body area compared with controls. Larval weight decreased from 0.37 ± 0.02 mg in controls to 0.25 ± 0.02 mg in LC_30_ and 0.20 ± 0.02 mg in LC_50_. Similarly, larval body area declined from 1.36 ± 0.08 mm^2^ in controls to 0.95 ± 0.08 mm^2^ in LC_30_ and 0.82 ± 0.08 mm^2^ in LC_50_. In contrast, neither the duration of the larval stage nor that of the pupal stage differed significantly among treatments. Pupation and adult emergence rates showed a decreasing trend with increasing *B. thuringiensis* INTA Mo4-4 concentration (adult emergence: 42.79% in controls; 26.29% in LC_30_; 11.78% in LC_50_), although these differences did not reach statistical significance (*p* > 0.05) due to high individual variability. Likewise, no significant differences were detected in pupal area, pupal weight, adult weight, or adult body area across treatment groups.

### 3.3. Sex-Specific Effects

Pupal and adult measurements disaggregated by sex are presented in Table 3. While statistical analysis (Bonferroni test) showed no significant differences in pupal or adult weight and area among treatments within each sex, there was a mild increase in female pupal weight and area at LC_30_ and LC_50_ compared to the control. Although non-significant (*p* > 0.05), this trend suggests a potential differential physiological response between genders under Bt stress.

As shown in Table 3, the effects of sublethal concentrations during the pupal and adult stages were influenced by gender. In pupae, female weight and body area tended to increase slightly at LC_30_ and LC_50_ compared with the control, whereas male pupae exhibited only minor, non-significant changes. Similarly, in adults, females showed a slight increase in weight and body area under LC_30_, while males displayed no significant variation across treatments. Statistical analysis using the Bonferroni test indicated that most of these differences were not significant at the 0.05 level, highlighting subtle, gender-specific responses.

### 3.4. Macromolecular Content

Biochemical analysis of surviving larvae (14 days post-exposure) is presented in Table 4. Proteins and lipids were the most sensitive reserves, showing significant reductions when expressed per individual in both LC_30_ and LC_50_ groups compared to the control (*p* < 0.05). Conversely, the contents of soluble sugars and glycogen did not differ significantly across treatments at the evaluated concentrations (Table 4).

Values in µg/larva; mean ± standard error; *n* = number of larvae per pool; means with same letter not significantly different, *p* > 0.05. The sample size (*n*) is variable as the colorimetric reactions were performed on surviving residual specimens from each bioassay date. Glycogen determinations include a higher number of replicates than glucose due to the exclusion of some glucose measurements that did not meet quality control criteria.

The data reveal that proteins and lipids are the most sensitive macromolecular targets of initial sublethal exposure. The significant decrease in these reserves is consistent with a high energetic cost associated with the immune response or the repair of intestinal damage caused by *B. thuringiensis* INTA Mo4-4. As shown in Table 4, the depletion of these energy-dense molecules was concentration-dependent, highlighting the metabolic stress imposed by the entomopathogenic bacteria during the first 14 days of exposure.

### 3.5. Long-Term Survival and Lethal Time (LT) Analysis

**Figure 1:**
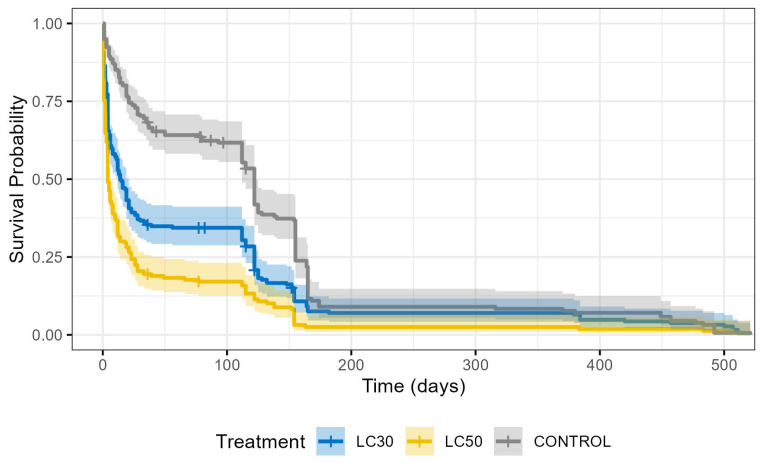
Kaplan-Meier survival curves indicating survival probability as a function of time. The three treatments under study were: CONTROL, LC_30_ and LC_50_. The shaded areas represent the 95% confidence intervals for each survival curve. Cross marks on the curves indicate censored observations.

The effects of *B. thuringiensis* INTA Mo4-4 treatment on the survival probability of *A. diaperinus* larvae over time are presented in Figure 1. Day 0 represents the end of the initial bioassay under moist diet conditions and the transition to individual dry-diet tubes. Individual survival records by insect, bioassay date, and treatment are available in the Supplementary Material (Table S5: Global survival analysis).

To maintain the rigor of the Kaplan-Meier analysis, individual specimens that could not be monitored until natural death—due to fungal contamination (non-Bt related) or technical incidents (e.g., escapes)—were treated as censored observations, as indicated by cross marks in Figure 1.

A statistically significant difference in survival was found among the treatment groups (χ^2^ = 109, df = 2, *p* < 2 × 10^−16^). The high χ^2^ value suggests a strong dose-dependent impact of the Argentine Bt strain on the longevity of the population. Significant differences between all pairs (Control vs. LC_30_, Control vs. LC_50_, and LC_30_ vs. LC_50_) were confirmed through pairwise comparisons adjusted with the Bonferroni method (*p* < 0.05).

Survival percentiles, including the median survival (LT_50_) and the time to 90% mortality (LT_90_), are summarized in Table 5. The marked effect of the INTA Mo4-4 strain is evident as the LT_50_ decreased dramatically from 116.58 days in the control to 14.28 and 4.19 days for LC_30_ and LC_50_, respectively. This indicates that even concentrations designed to be sublethal in the short term trigger chronic lethal effects with an accelerated mortality rate shortly after ingestion.

Furthermore, the chronic nature of these effects is reflected in the LT_90_ values. Although a small fraction of the population exhibited high resilience and a prolonged lifespan, the time required to reach 90% mortality was reduced by approximately 24% and 40% in the LC_30_ and LC_50_ groups, respectively, compared to the control. This confirms that the physiological impairment sustained during the larval stage results in persistent biological costs that significantly shorten the maximum life expectancy of the species.

## 4. Discussion

This study investigated the initial sublethal effects of the Argentine *Bacillus thuringiensis* strain INTA Mo4-4 on the development, fitness, and nutritional physiology of *Alphitobius diaperinus* larvae. The interpretation of the observed effects is based on bioassay evidence rather than on direct molecular or proteomic identification of individual Cry, Cyt, Vip, or Vpa/Vpb proteins produced by INTA Mo4-4. Accordingly, the precise virulence factors involved were not identified in the present study, and references to Cry toxins should be understood within this experimental context. The results demonstrate profound and persistent chronic effects, particularly impacting larval growth, survival, and energy reserves. These findings highlight the potential of initial sublethal *B. thuringiensis* concentrations to disrupt the life cycle and fitness of this major poultry pest, offering insights for integrated pest management (IPM) strategies.

Although the present bioassays were conducted under controlled laboratory conditions, with larvae maintained individually to prevent cannibalism, these constraints do not preclude the ecological relevance of the observed effects. Under field conditions, where food limitation and high larval densities may occur, exposure to *B. thuringiensis* could be extended through indirect pathways such as cannibalism, a transmission route previously demonstrated in tenebrionid beetles [25]. Such processes may contribute to sustained sublethal exposure and reinforce the persistence of chronic effects in natural populations.

Consistent with this complexity, *A. diaperinus* exhibited pronounced intraspecific variability both within and among cohorts, reflecting the well-known resilience of tenebrionid beetles. Cohorts, defined here as individuals hatching within a 24–48 h oviposition window, showed marked developmental asynchrony despite standardized rearing conditions. Notably, within the same cohort and treatment, the interval between the first and last individuals reaching pupation extended up to 86 days (72 to 158 days), and younger cohorts occasionally pupated earlier than older ones. Across bioassays, this variability was expressed as contrasting developmental outcomes (additional information is provided in the Supplementary Materials) ranging from cases in which only control individuals completed development to adulthood, to others in which both control and LC_30_ larvae reached the adult stage, and, in some instances, survivors from all three treatments emerged as adults. When survival occurred across treatments, two distinct patterns were observed: either larval development converged toward control-like durations (LC_50_ ≈ LC_30_ ≈ control), or treated larvae required longer developmental times than controls (LC_50_ > LC_30_ > control).

This high developmental plasticity, together with physiological traits characteristic of beetle larvae—such as an acidic midgut environment that may limit crystal solubilization and reduce Cry toxin activation [26,27]—likely contributes to the wide variability observed in survival and developmental endpoints. Importantly, even in cases where apparent developmental recovery was observed, sublethal effects persisted, as evidenced by delayed mortality patterns reflected in LT_50_ and LT_90_ estimates, underscoring the chronic nature of the effects detected. The reductions in larval weight, body area, and total protein and lipid content observed after 14 days of exposure may result from reduced nutrient intake and/or impaired nutrient absorption. Given the drastic reduction in LT_50_ observed in treated larvae, the LC_30_ and LC_50_ used here are best interpreted as chronic lethal concentrations. The depletion of protein and lipid reserves serves as a biochemical proxy for the energetic costs of surviving initial intoxication. While we recognize the absence of food consumption measurements or gut histology as a limitation, the significant reduction in these energy-dense macromolecules suggests a metabolic trade-off, where energy is diverted from growth toward detoxification or repair of intestinal damage. Nevertheless, although food consumption and frass production were not directly quantified in this study, we cannot rule out the possibility that larvae may have reduced their feeding activity. Similar patterns have been documented in other species. Sutherland et al., 2003 [28] reported that starvation in *Epiphyas postvittana* (Walker) (Lepidoptera: Tortricidae) larvae decreased midgut cell size without causing lysis, and that individuals fed a Cry1Ac diet showed a feeding recovery but ultimately reached a mean weight comparable to starved larvae.

Likewise, Luong et al., 2018 [29] suggested that behavioral avoidance of the toxin contributed to the survival of *Helicoverpa armigera* (Hübner) (Lepidoptera: Noctuidae) on Bt-expressing plants, and Berdegué et al., 1996 [30] demonstrated significant avoidance of Bt-treated diet by neonatal and third-instar *Spodoptera exigua* (Hübner) (Lepidoptera: Noctuidae), with larvae consuming substantially more control diet across CryIC treatments. Together, these findings support the possibility that reduced feeding—whether due to behavioral avoidance or physiological stress—may contribute to the nutritional depletion observed in our study.

Although the specific molecular identity of the pesticidal proteins in INTA Mo4-4 is currently being elucidated via genomic sequencing, the localization of toxicity within the spore-crystal pellet aligns with the typical pathology of *B. thuringiensis* in coleopterans. The chronic effects observed here—reduced body mass and delayed mortality—suggest a disruption of midgut integrity. In Tenebrionidae, this usually involves the binding of Cry toxins to specific epithelial receptors, leading to septicemia or functional starvation [7,12,13].

In coleopteran insects, the mode of action of *B. thuringiensis* Cry toxins has been closely associated with specific midgut receptors, particularly cadherins. In *A. diaperinus*, Hua et al., 2014 identified the cadherin AdCad1 as a specific receptor for the Cry3Bb toxin in larval midgut cells [12]. Subsequently, Park et al., 2014 demonstrated that a fragment of the coleopteran cadherin DvCad1-CR8–10 synergistically enhances the toxicity of Cry3Aa, Cry3Bb, and Cry8Ca, highlighting the functional role of cadherin-mediated toxin binding in this species [7]. Although the present study did not directly investigate Bt–receptor interactions or cadherin involvement, these previously described mechanisms may contribute to, or be associated with, the biological effects observed here. In line with this mechanistic framework, the patterns observed in our study are also compatible with the well-documented disruption of the midgut epithelium caused by *Bacillus thuringiensis* Cry toxins, which can compromise digestive efficiency and lead to nutritional depletion. A substantial body of evidence supports this mechanism: Heckel 2020 [31] highlights pore formation in epithelial membranes as the primary cause of Cry-induced cytotoxicity, with ABC transporters and cadherins acting as key receptors whose disruption severely alters gut integrity. Consistently, Bowling et al., 2017 [32] reported clear signs of intoxication in *Diabrotica virgifera virgifera* LeConte (Coleoptera: Chrysomelidae) larvae exposed to various insecticidal proteins, including swelling and sloughing of enterocytes and constriction of midgut circular muscles. More recently, Ayra-Pardo et al., 2025 [33] showed that Cry1Ia-fed *Rhynchophorus ferrugineus* Olivier (Coleoptera: Curculionidae) larvae exhibited extensive midgut cell damage, impairment of digestion and nutrient absorption, and loss of the peritrophic membrane. Together, these studies provide a coherent physiological explanation for the nutritional depletion recorded in our bioassays.

Within the framework of the standardized bioassay, LC_30_ and LC_50_ correspond to concentrations at which a substantial proportion of individuals survive the initial exposure period, while delayed mortality is revealed only through extended monitoring. Under these conditions, approximately 70% and 50% of larvae survived the 14-day exposure to LC_30_ and LC_50_, respectively, and only surviving individuals were subsequently followed. The limited acute mortality observed during exposure likely reflects individual variability in susceptibility and behavioral responses to *B. thuringiensis*, including transient reductions in feeding that may temporarily limit toxin ingestion. However, the pronounced, dose-dependent reductions in LT_50_ values and the delayed mortality observed after transfer to a toxin-free diet indicate that initial sublethal exposure induces chronic physiological damage that is not fully expressed as acute mortality within standard short-term bioassays. Thus, these concentrations, while permitting survival during the initial 14-day assay, should be interpreted as chronic lethal doses due to the irreversible energetic and physiological damage sustained by the larvae.

### 4.1. Impact on Larval Growth and Metabolism

The most significant sublethal effects observed were the severe reduction in larval weight and body area (Table 2) and the dramatic depletion of key macromolecular reserves (Table 4). The reduction in larval mass, which was proportional to the *B. thuringiensis* concentration, is a classic sign of intoxication by Cry proteins. Upon ingestion, Cry toxins cause pore formation in the midgut epithelial cells, disrupting osmotic balance and nutrient absorption [34,35]. The larvae likely compensated for this midgut damage and nutrient malabsorption by diverting limited energy resources away from somatic growth towards tissue repair, detoxification, and stress management, resulting in smaller body size. The analysis of macromolecular content reinforces this interpretation. Protein content experienced the most severe depletion, followed by lipids. Proteins are crucial for cellular maintenance, enzyme synthesis, and growth [36,37]. Their loss suggests severe tissue damage and/or a failure in synthesizing new proteins, possibly due to reduced energy input or direct Cry action on the gut [38]. The significant loss of lipids, one of the primary long-term energy reserves and an important contributor to insect immune function, indicates that larvae metabolized their reserves to fuel basic survival functions and repair gut damage [28,39]. Importantly, the observed reductions in protein and lipid content are consistent with metabolic stress and/or reduced nutrient intake but are not presented here as direct evidence of immune activation or gut repair processes.

In *A. diaperinus*, total sugars and glycogen are rarely quantified, likely because they represent minor components relative to total proteins and lipids. Based on findings in other beetles where sugars are depleted during food deprivation [40,41], we hypothesize that larvae surviving 14 days of exposure may have experienced a combination of self-imposed fasting and/or impaired nutrient absorption. In our bioassays, no statistically significant differences were detected in total sugars or glycogen, likely due to high internal variability; nevertheless, trends were consistent with this physiological framework. These biochemical disruptions are highly correlated with the impaired larval growth [42] (Table 2).

### 4.2. Chronic Effects on Development and Fitness

Despite the severe physiological stress, the duration of the larval stage did not change significantly (Table 2). This lack of developmental delay, despite being smaller and metabolically stressed, contrasts with studies in other insects [43,44] and suggests that the surviving *A. diaperinus* may have accelerated their development as a stress response to quickly exit the toxic environment. However, this rapid development came at a clear cost to overall fitness, as evidenced by the sharp, though statistically non-significant, decrease in pupation and adult emergence rates (Table 2).

The most striking long-term impact was the catastrophic reduction in survival time (Figure 1, Table 5). The LT_50_ for the LC_50_ group was reduced from over 116 days to just 4 days, indicating that the chronic physiological damage sustained was severe enough to drastically shorten the life expectancy of the surviving population. This finding underscores the concept that sublethal exposure can act as a delayed mortality factor, a highly effective result in pest control where persistent suppression is key [45,46,47].

### 4.3. Gender-Specific Responses

Females of *A. diaperinus* tend to have larger body size than males, an advantage for tolerating desiccation [48]. The absence of significant differences in final pupal and adult body size suggests that survivors achieved a final size similar to the control group, potentially through compensatory growth. However, Table 3 hints at subtle gender-specific responses. While not statistically significant, female pupal and adult sizes trended slightly higher in the LC_30_ group. This phenomenon, where females exhibit a positive size trend under mild stress, may be related to sex-specific resource allocation for egg production [49,50].

### 4.4. Deformations by B. thuringiensis

Several cases of deformities induced by *B. thuringiensis* have been reported in species such as *Drosophila melanogaster* Meigen (Diptera: Drosophilidae) [51], *Galleria mellonella* (Linnaeus) (Lepidoptera: Pyralidae) [52] and *Anastrepha fraterculus* (Wiedemann) (Diptera: Tephritidae) [53]. To the best of our knowledge, no reports have documented deformities in beetles associated with *B. thuringiensis* exposure.

After extensive bioassays with INTA Mo4-4, we found no macroscopic evidence of teratogenic effects in *A. diaperinus*. Notably, developmental damage was occasionally observed in control specimens during molting, a physiologically stressful phase. Although alterations at the cellular level may occur—as revealed by micro-CT in *Aedes aegypti* (Linnaeus) (Diptera: Culicidae) [54]—our conclusions are limited to the absence of macroscopic deformities.

### 4.5. Implications for Pest Management

The results from this study confirm that the *B. thuringiensis* INTA Mo4-4 strain has significant potential for *A. diaperinus* control through both acute toxicity and potent sublethal effects. Exposure to LC_30_ and LC_50_ concentrations leads to: reduced larval growth affecting subsequent reproductive capacity; severe metabolic stress compromising long-term survival; and delayed yet high mortality.

These sublethal effects are highly relevant to field conditions in poultry houses, where uneven application may result in larvae consuming non-lethal doses. The chronic toxicity observed suggests that *B. thuringiensis* applications do not need to achieve 100% mortality to be highly effective; instead, they function as potent growth and fitness suppressors. Incorporating INTA Mo4-4 into an IPM program could therefore offer sustained pest suppression by reducing the next generation’s population size and overall lifespan.

## 5. Conclusions

Initial sublethal exposure to *Bacillus thuringiensis* INTA Mo4-4 induced profound and persistent physiological stress in *Alphitobius diaperinus*, with consequences extending far beyond the initial exposure period. Larvae that survived the 14-day sublethal bioassays exhibited marked reductions in body size, weight, and key macromolecular reserves, particularly proteins and lipids, indicating severe impairment of growth and metabolic homeostasis. These alterations reflect a state of chronic toxicity that likely compromises the ability of individuals to cope with subsequent environmental challenges and may negatively affect future reproductive performance.

These effects were supported by statistically significant differences detected at multiple biological levels. Significant reductions in larval area and weight were observed when comparing both sublethal treatments (LC_30_ and LC_50_) with controls. In terms of survival, the overall survival analysis revealed a significant effect of treatment, and subsequent pairwise comparisons showed significant differences between all treatment combinations (control vs. LC_30_, control vs. LC_50_, and LC_50_ vs. LC_30_). In addition, the nutritional composition of *A. diaperinus* larvae differed significantly between controls and both treatments after 14 days of dietary exposure, specifically in the total individual content of proteins and lipids.

Importantly, the physiological damage sustained during sublethal exposure translated into pronounced delayed mortality, as evidenced by the drastic reduction in LT_50_ and LT_90_ values even after larvae were transferred to an uncontaminated diet. This demonstrates that initial sublethal doses of *B. thuringiensis* can act as a powerful delayed-lethal factor, substantially shortening lifespan and reducing population persistence despite apparent short-term survival or partial developmental recovery.

Collectively, our results show that *B. thuringiensis* INTA Mo4-4 exerts its insecticidal activity against *A. diaperinus* not only through direct lethality but also through sustained chronic sublethal effects that disrupt growth, metabolism, survival, and overall fitness across the life cycle. Although the present study does not include molecular or proteomic characterization of individual Cry, Cyt, Vip, or Vpa/Vpb proteins and therefore does not aim to dissect the specific mechanisms of action of *B. thuringiensis* in coleopterans, our interpretation—grounded in previous reports and consistent with the entomological scope of this work—supports two non-exclusive hypotheses, namely larval self-imposed starvation as a survival strategy and Bt-induced midgut damage, and leaves no doubt that *B. thuringiensis* INTA Mo4-4 induces chronic toxicity and delayed mortality. From an applied perspective, these characteristics make INTA Mo4-4 a strong candidate for the biocontrol of beetle pests, either as a standalone tool or as part of an integrated pest management program. The ability to suppress populations through chronic toxicity and delayed mortality is particularly relevant under field conditions, where exposure to non-lethal doses is common. While the molecular identification of its pesticidal proteins is currently underway, the consistent and long-term impact on *A. diaperinus* development and survival reported here validates this strain as a solid biocontrol agent for the development of sustainable insecticides.

## Figures and Tables

**Table 1:** Sublethal concentrations of a spore-crystal suspension of *B. thuringiensis* INTA Mo4-4 against second-instar larvae of *A. diaperinus* 14 days post treatment.

Assay	LC_30_ ^1^ (µg/mL)	LC_50_ ^1^ (µg/mL)	Slope ^3^	χ^2^ (4 df) ^4^
1	67.76 [52.64–81.64]	117.83 [99.24–140.86]	2.18	3.97
2	84.34 [65.65–102.24]	154.55 [128.08–194.19]	1.99	1.85
3	61.98 [40.35–81.21]	144.87 [113.21–198.76]	1.42	4.58
4	61.55 [19.66–95.36]	125.69 [77.50–229.18]	1.69	7.88
Mean CV ^2^	68.91 15.49%	135.74 12.46%		

LC_30_ ^1^ and LC_50_ ^1^ (lethal concentration) average of four repetitions + 95% confidence limits for concentration; ^2^ coefficient of variation; ^3^ slope; ^4^ χ^2^ with four degrees of freedom (df).

**Table 2:** Sublethal effects of LC_30_ and LC_50_ on biological parameters of *A. diaperinus*. The average, minimum, and maximum values ± standard error (S.E.) are indicated for each parameter.

Variable	Control (Mean ± S.E. [min–max])	*n*	LC_30_ (Mean ± S.E. [min–max])	*n*	LC_50_ (Mean ± S.E. [min–max])	*n*
Larval weight (mg)	0.37 ^B^ ± 0.02 [0.28–0.42]	192	0.25 ^A^ ± 0.02 [0.23–0.28]	180	0.20 ^A^ ± 0.02 [0.17–0.22]	178
Larval area (mm^2^)	1.36 ^B^ ± 0.08 [1.17–1.67]	192	0.95 ^A^ ± 0.08 [0.87–1.06]	180	0.82 ^A^ ± 0.08 [0.71–0.95]	178
Larval stage duration from hatching (days)	94.69 ^A^ ± 9.17 [78.31–105.00]	106	101.13 ^A^ ± 10.59 [80.92–114.48]	71	100.65 ^A^ ± 12.97 [78.70–122.60]	28
Larval stage duration since the end of Bt intake (days)	76.69 ^A^ ± 9.17 [60.31–87.00]	106	83.13 ^A^ ± 10.59 [62.92–96.48]	71	82.65 ^A^ ± 12.97 [60.70–104.60]	28
Pupation rate (%)	42.79 ^A^ ± 15.17 [4.17–81.25]	106	26.58 ^A^ ± 15.17 [0.00–54.55]	71	11.78 ^A^ ± 15.17 [0.00–32.39]	28
Pupal stage duration (days)	5.71 ^A^ ± 0.30 [5.00–6.36]	95	6.35 ^A^ ± 0.35 [5.93–6.62]	65	6.95 ^A^ ± 0.43 [6.29–7.60]	26
Pupal area (mm^2^)	10.21 ^A^ ± 1.27 [9.32–11.10]	87	9.78 ^A^ ± 1.04 [8.44–11.21]	59	9.74 ^A^ ± 1.27 [7.80–11.68]	26
Pupal weight (mg)	11.30 ^A^ ± 1.74 [10.10–12.50]	87	10.95 ^A^ ± 1.42 [8.75–13.05]	59	10.58 ^A^ ± 1.74 [8.12–13.04]	26
Adults rate (%)	42.79 ^A^ ± 15.11 [4.17–81.25]	106	26.29 ^A^ ± 15.11 [0.00–53.41]	70	11.78 ^A^ ± 15.11 [0.00–32.39]	28
Adult area (mm^2^)	9.87 ^A^ ± 0.92 [8.70–11.28]	80	9.72 ^A^ ± 0.92 [8.54–11.38]	59	9.52 ^A^ ± 1.13 [7.95–11.08]	23
Adult weight (mg)	9.48 ^A^ ± 1.13 [8.42–11.04]	80	9.65 ^A^ ± 1.13 [7.65–11.35]	59	9.01 ^A^ ± 1.39 [6.96–11.06]	23

Means with a common letter are not significantly different (*p* > 0.05). The variation in *n* observed among treatments and recorded variables reflects the progressive decline in the number of surviving specimens over time, primarily due to treatment effects. In addition, some individuals escaped from their individual containers and became mixed, and others were lost because their diet became compromised by fungal growth. These specimens were excluded from the sublethal dataset throughout the experiments.

**Table 3:** Sublethal effects on pupal and adult biological parameters with gender interaction.

Stage	Variable	Gender	Control (Mean ± S.E. [min–max])	*n*	LC_30_ (Mean ± S.E. [min–max])	*n*	LC_50_ (Mean ± S.E. [min–max])	*n*
Pupae	Weight	Female	11.95 ^AB^ ± 0.56 [8.18–17.83]	24	12.77 ^B^ ± 0.62 [8.11–19.76]	20	12.35 ^AB^ ± 1.23 [9.27–16.10]	5
		Male	9.30 ^A^ ± 0.60 [6.85–15.47]	21	9.47 ^A^ ± 0.67 [4.44–15.00]	17	9.62 ^AB^ ± 0.97 [7.54–14.09]	8
	Area	Female	10.13 ^BC^ ± 0.39 [7.20–14.80]	24	10.73 ^BC^ ± 0.42 [7.90–13.50]	20	11.34 ^C^ ± 0.85 [9.20–13.50]	5
		Male	8.61 ^A^ ± 0.41 [5.90–12.00]	21	8.90 ^A^ ± 0.46 [5.70–13.70]	17	9.20 ^AB^± 0.67 [6.60–12.70]	8
Adult	Weight	Female	10.45 ^AB^ ± 0.47 [6.97–14.18]	24	11.19 ^B^ ± 0.54 [6.87–16.71]	18	10.96 ^AB^ ± 1.02 [8.34–13.82]	5
		Male	8.22 ^A^ ± 0.54 [7.76–12.91]	18	8.03 ^A^ ± 0.55 [3.47–12.34]	17	7.98 ^A^ ± 0.81 [5.67–12.21]	8
	Area	Female	10.69 ^AB^ ± 0.39 [7.80–15.40]	24	11.38 ^B^ ± 0.46 [7.70–16.00]	18	10.34 ^AB^ ± 0.86 [7.80–12.40]	5
		Male	8.97 ^A^ ± 0.46 [6.50–12.40]	18	9.05 ^A^ ± 0.47 [4.90–11.00]	17	9.06 ^AB^ ± 0.68 [6.90–12.70]	8

Means with a common letter are not significantly different (*p* > 0.05).

**Table 4:** Macromolecule content of *A. diaperinus* larvae under control, LC_30_ and LC_50_ treatments.

Macromolecule	Control (Mean ± S.E.) [min–max]	*n*	LC_30_ (Mean ± S.E.) [min–max]	*n*	LC_50_ (Mean ± S.E.) [min–max]	*n*
Proteins	13.23 ^B^ ± 1.03 [10.43–17.13]	23	6.36 ^A^ ± 1.19 [5.46–7.75]	20	3.76 ^A^ ± 1.03 [2.67–6.00]	35
Lipids	19.87 ^B^ ± 1.63 [14.04–27.56]	48	11.04 ^A^ ± 2.30 [8.37–13.31]	30	9.36 ^A^ ± 1.99 [6.22–13.64]	40
Sugars	2.14 ^A^ ± 0.73 [0.89–3.38]	24	1.90 ^A^ ± 0.73 [1.66–2.14]	24	1.66 ^A^ ± 0.73 [1.63–1.68]	24
Glycogen	0.13 ^A^ ± 0.03 [0.07–0.20]	69	0.12 ^A^ ± 0.03 [0.10–0.13]	49	0.07 ^A^ ± 0.03 [0.03–0.13]	44

Means with a common letter are not significantly different (*p* > 0.05).

**Table 5:** Survival percentiles for each treatment group (days).

Treatment Group	LT_50_ (CI)	LT_90_ (CI)
Control	116.58 (106.95–121.35)	218.70 (164.63–427.88)
LC_30_	14.28 (10.78–18.92)	166.80 (149.85–331.07)
LC_50_	4.19 (3.43–6.00)	130.70 (112.85–152.54)

LT_50_ = median lethal time; LT_90_ = time at which 90% of individuals have died; CI = 95% confidence interval.

## Data Availability

Data are contained within the article.

## Supplementary Materials

The following supporting information can be downloaded at: https://www.mdpi.com/article/10.3390/insects17020213/s1, Table S1: Individual larval weight; Table S2: Individual larval area; Table S3: Individual larval and pupal stage duration; Table S4: Pupae and adults area and weight; Table S5: Global survival analysis.

## Abbreviations

The following abbreviations are used in this manuscript:

LC	Lethal Concentration
LT	Lethal Time
IPM	Integrated Pest Management

## References

[1] Panzer G.W.F. (1793). Faunae Insectorum Germanicae Initia Oder Deutschlands Insecten.

[2] Goodwin M.A., Waltman W.D. (1996). Transmission of Eimeria, Viruses, and Bacteria to Chicks: Darkling Beetles (*Alphitobius diaperinus*) as Vectors of Pathogens. J. Appl. Poult. Res..

[3] Tufan-Cetin O., Cetin H.A. (2025). Review of Biological and Sustainable Management Approaches for *Alphitobius diaperinus*, a Major Pest in Poultry Facilities. Vet. Sci..

[4] Sammarco B.C., Hinkle N.C., Crossley M.S. (2023). Biology and Management of Lesser Mealworm *Alphitobius diaperinus* (Coleoptera: Tenebrionidae) in Broiler Houses. J. Integr. Pest Manag..

[5] Crickmore N., Berry C., Panneerselvam S., Mishra R., Connor T.R., Bonning B.C. (2021). A structure-based nomenclature for *Bacillus thuringiensis* and other bacteria-derived pesticidal proteins. J. Invertebr. Pathol..

[6] Krieg A., Huger A.M., Langenbruch G.A., Schnetter W. (1983). *Bacillus thuringiensis* var. *tenebrionis*: Ein neuer, gegenüber Larven von Coleopteren wirksamer Pathotyp. Z. Angew. Entomol..

[7] Park Y., Hua G., Taylor M.D., Adang M.J. (2014). A coleopteran cadherin fragment synergizes toxicity of *Bacillus thuringiensis* toxins Cry3Aa, Cry3Bb, and Cry8Ca against lesser mealworm. *Alphitobius diaperinus* (Coleoptera: Tenebrionidae). J. Invertebr. Pathol..

[8] Sallet L. (2013). Seleção de Estirpes de *Bacillus thuringiensis* Para o Controle de *Alphitobius diaperinus* (Coleoptera: Tenebrionidae). Ph.D. Thesis.

[9] Pérez M.P., Benintende G.B., Sauka D.H. (2025). Toxicity assessment of *Bacillus thuringiensis* strains for the control of the lesser mealworm beetle *Alphitobius diaperinus* (Coleoptera: Tenebrionidae). Rev. Argent. Microbiol..

[10] Koc S., Polat B., Cengiz A., Kahraman S., Tufan Cetin O., Cetin H. (2022). Effectiveness of some microbial biopesticides based on *Bacillus* against lesser mealworm *Alphitobius diaperinus* (Coleoptera: Tenebrionidae) under laboratory conditions. Fresenius Environ. Bull..

[11] Elgizawy K.K., Ashry N.M. (2019). Efficiency of *Bacillus thuringiensis* strains and their Cry proteins against the Red Flour Beetle, *Tribolium castaneum* (Herbst.) (Coleoptera: Tenebrionidae). Egypt. J. Biol. Pest Control.

[12] Hua G., Park Y., Adang M.J. (2014). Cadherin AdCad1 in *Alphitobius diaperinus* larvae is a receptor of Cry3Bb toxin from *Bacillus thuringiensis*. Insect Biochem. Mol. Biol..

[13] Hasan M.M., Parween S., Reza A.M.S., Easmin N. (2002). Response of *Alphitobius diaperinus* Panzer to *Bacillus thuringiensis* Berliner var. *kurstaki*. Entomon.

[14] Behle R.W., McGuire M.R., Shasha B.S. (1997). Effects of Sunlight and Simulated Rain on Residual Activity of *Bacillus thuringiensis* Formulations. J. Econ. Entomol..

[15] Pérez M.P. (2017). Factores de Virulencia de *Bacillus thuringiensis* y su Utilización Para el Control de Coleópteros de Alto Impacto en el Sector Agropecuario. Ph.D. Thesis.

[16] Iriarte J., Caballero P., Caballero P., Ferré J., Del Río J.L. (2001). Biología y Ecología de *Bacillus thuringiensis*. Bioinsecticidas: Fundamentos y Aplicaciones de Bacillus thuringiensis en el Control Integrado de Plagas.

[17] Finney D.J. (1971). Probit Analysis.

[18] Nouri-Ganbalani G., Borzoui E., Abdolmaleki A., Abedi Z., Kamita S.G. (2016). Individual and Combined Effects of *Bacillus Thuringiensis* and Azadirachtin on *Plodia Interpunctella* Hübner (Lepidoptera: Pyralidae). J. Insect Sci..

[19] Abedi Z., Saber M., Vojoudi S., Mahdavi V., Parsaeyan E. (2014). Acute, sublethal, and combination effects of azadirachtin and *Bacillus thuringiensis* on the cotton bollworm, *Helicoverpa armigera*. J. Insect Sci..

[20] Esquivel J.F., Crippen T.L., Ward L.A. (2012). Improved Visualization of *Alphitobius diaperinus* (Panzer) (Coleoptera: Tenebrionidae)—Part I: Morphological Features for Sex Determination of Multiple Stadia. Psyche A J. Entomol..

[21] Van Herk W.G., Vernon R.S., Tolman J.H., Ortiz Saavedra H. (2008). Mortality of a wireworm, *Agriotes obscurus* (Coleoptera: Elateridae), after topical application of various insecticides. J. Econ. Entomol..

[22] Brogdon W.G. (1984). Mosquito protein microassay—I. Protein determinations from small portions of single-mosquito homogenates. Comp. Biochem. Physiol. B.

[23] Anschau A., Caruso C.S., Kuhn R.C., Franco T.T. (2017). Validation of the sulfo-phospho-vanillin (SPV) method for the determination of lipid content in oleaginous microorganisms. Braz. J. Chem. Eng..

[24] Yuval B., Kaspi R., Shloush S., Warburg M.S. (1998). Nutritional reserves regulate male participation in Mediterranean fruit fly leks. Ecol. Entomol..

[25] Milutinović B., Höfling C., Futo M., Scharsack J.P., Kurtz J. (2015). Infection of *Tribolium castaneum* with *Bacillus thuringiensis*: Quantification of Bacterial Replication within Cadavers, Transmission via Cannibalism, and Inhibition of Spore Germination. Appl. Environ. Microbiol..

[26] Dow J.A.T., Evans P.D., Wigglesworth V.B. (1987). Insect midgut function. Advances in Insect Physiology.

[27] Oppert B. (1999). Protease interactions with *Bacillus thuringiensis* insecticidal toxins. Arch. Insect Biochem. Physiol..

[28] Sutherland P.W., Harris M.O., Markwick N.P. (2003). Effects of Starvation and the *Bacillus thuringiensis* Endotoxin Cry1Ac on the Midgut Cells, Feeding Behavior, and Growth of Lightbrown Apple Moth Larvae. Ann. Entomol. Soc. Am..

[29] Luong T.T.A., Zalucki M.P., Perkins L.E., Downes S.J. (2018). Feeding behaviour and survival of *Bacillus thuringiensis*-resistant and *Bacillus thuringiensis*-susceptible larvae of *Helicoverpa armigera* (Lepidoptera: Noctuidae) exposed to a diet with *Bacillus thuringiensis* toxin. Austral Entomol..

[30] Berdegué M., Trumble J.T., Moar W.J. (1996). Effect of CryIC toxin from *Bacillus thuringiensis* on larval feeding behavior of *Spodoptera exigua*. Entomol. Exp. Appl..

[31] Heckel D.G. (2020). How do toxins from *Bacillus thuringiensis* kill insects? An evolutionary perspective. Arch. Insect Biochem. Physiol..

[32] Bowling A.J., Pence H.E., Li H., Tan S.Y., Evans S.L., Narva K.E. (2017). Histopathological effects of Bt and TcdA insecticidal proteins on the midgut epithelium of Western corn rootworm larvae (*Diabrotica virgifera virgifera*). Toxins.

[33] Ayra-Pardo C., Ramare V., Couto A., Almeida M., Martins R., Sousa J.A., Santos M.J. (2025). The Proteolytic Activation, Toxic Effects, and Midgut Histopathology of the *Bacillus thuringiensis* Cry1Ia Protoxin in *Rhynchophorus ferrugineus* (Coleoptera: Curculionidae). Toxins.

[34] Palma L., Muñoz D., Berry C., Murillo J., Caballero P. (2014). *Bacillus thuringiensis* toxins: An overview of their biocidal activity. Toxins.

[35] Melo A.L.A., Soccol V.T., Soccol C.R. (2014). *Bacillus thuringiensis*: Mechanism of action, resistance, and new applications: A review. Crit. Rev. Biotechnol..

[36] Corsetti G., Pasini E., Scarabelli T.M., Romano C., Singh A., Scarabelli C.C., Dioguardi F.S. (2024). Importance of Energy, Dietary Protein Sources, and Amino Acid Composition in the Regulation of Metabolism: An Indissoluble Dynamic Combination for Life. Nutrients.

[37] Kurečka M., Kulma M., Petříčková D., Plachý V., Kouřimská L. (2021). Larvae and pupae of *Alphitobius diaperinus* as promising protein alternatives. Eur. Food Res. Technol..

[38] Khatami L., Ghassemi-Kahrizeh A., Hosseinzadeh A., Aramideh S. (2023). The effect of sublethal doses of *Bacillus thuringiensis* Berliner on *Tuta absoluta* (Meyrick) on resistant and susceptible tomato cultivars. Zemdirbyste-Agriculture.

[39] Arrese E.L., Soulages J.L. (2010). Insect fat body: Energy, metabolism, and regulation. Annu. Rev. Entomol..

[40] Laparie M., Larvor V., Frenot Y., Renault D. (2012). Starvation resistance and effects of diet on energy reserves in a predatory ground beetle (*Merizodus soledadinus*; Carabidae) invading the Kerguelen Islands. Comp. Biochem. Physiol. A.

[41] Renault D., Hervant F., Vernon P. (2002). Comparative study of the metabolic responses during food shortage and subsequent recovery at different temperatures in the adult lesser mealworm, *Alphitobius diaperinus* (Coleoptera: Tenebrionidae). Physiol. Entomol..

[42] Chauhan V.K., Dhania N.K., Chaitanya R.K., Senthilkumaran B., Dutta-Gupta A. (2017). Larval Mid-Gut Responses to Sub-Lethal Dose of Cry Toxin in Lepidopteran Pest *Achaea janata*. Front. Physiol..

[43] Ghassemi-Kahrizeh A., Aramideh S. (2015). Sub-lethal effects of *Bacillus thuringiensis* Berliner on larvae of Colorado potato beetle, *Leptinotarsa decemlineata* (Say) (Coleoptera: Chrysomelidae). Arch. Phytopathol. Plant Prot..

[44] Eizaguirre M., Tort S., López C., Albajes R. (2005). Effects of sublethal concentrations of *Bacillus thuringiensis* on larval development of *Sesamia nonagrioides*. J. Econ. Entomol..

[45] Stenekamp D., Pringle K., Addison M. (2016). Effect of genetically modified Bt maize in an artificial diet on the survival of *Cydia pomonella* (Lepidoptera: Tortricidae). Fla. Entomol..

[46] Wang L.Y., Jaal Z. (2005). Sublethal effects of *Bacillus thuringiensis* H-14 on the survival rate, longevity, fecundity and F1 generation developmental period of *Aedes aegypti*. Dengue Bull..

[47] Fast P.G., Régnière J. (1984). Effect of exposure time to *Bacillus thuringiensis* on mortality and recovery of the spruce budworm (Lepidoptera: Tortricidae). Can. Entomol..

[48] Renault D., Coray Y. (2004). Water loss of male and female *Alphitobius diaperinus* (Coleoptera: Tenebrionidae) maintained under dry conditions. Eur. J. Entomol..

[49] Celi M., Russo D., Vazzana M., Arizza V., Manachini B. (2022). Does *Bacillus thuringiensis* Affect the Stress and Immune Responses of *Rhynchophorus ferrugineus* Larvae, Females, and Males in the Same Way?. Insects.

[50] Belousova I., Pavlushin S., Subbotina A., Rudneva N., Martemyanov V. (2021). Sex specificity in innate immunity of insect larvae. J. Insect Sci..

[51] Nawrot-Esposito M.P., Babin A., Pasco M., Poirié M., Gatti J.-L., Gallet A. (2020). *Bacillus thuringiensis* Bioinsecticides Induce Developmental Defects in Non-Target *Drosophila melanogaster* Larvae. Insects.

[52] Al-Mashhadani M.M.A., Al-Joboory R.K.I. (2022). Effect of *Bacillus thuringiensis* on the biological aspects of the great waxworm *Galleria mellonella*. Int. J. Health Sci..

[53] Martins L.N., de Souza Stori de Lara A.P., Ferreira M.S., Nunes A.M., Bernardi D., Leivas Leite F.P., Garcia F.R.M. (2018). Biological Activity of *Bacillus thuringiensis* (Bacillales: Bacillaceae) in *Anastrepha fraterculus* (Diptera: Tephritidae). J. Econ. Entomol..

[54] Alba-Tercedor J., Vilchez S. (2023). Anatomical damage caused by *Bacillus thuringiensis* variety *israelensis* in yellow fever mosquito *Aedes aegypti* (L.) larvae revealed by micro-computed tomography. Sci. Rep..

