# Supplementary material for: Initial Sublethal Exposure to an Argentine Bacillus thuringiensis Strain Induces Chronic Toxicity and Delayed Mortality in Alphitobius diaperinus (Coleoptera: Tenebrionidae)
Source: Insects. 2026 Feb 18;17(2):213. doi: 10.3390/insects17020213 (PMC12942231; doi:10.3390/insects17020213)
Supplement: Supplementary file 1 [file insects-17-00213-s001.zip › insects-4094132-supplementary.pdf]

insects

MDPI

Supplementary Information

Table S1: Individual larval weight

Treatment	Bioassay date	Average individual larval weight + (n)	Mean larval weight	Standard deviation	Coefficient of variation
CONTROL	December 5–19, 2023<br>January 1–15, 2024<br>January 17-31, 2024<br>January 27–February 10, 2024	0.41 (48)<br>0.28 (48)<br>0.37 (48)<br>0.42 (48)	0.37	0.06	16.80
LC_{30}	December 5–19, 2023<br>January 1–15, 2024<br>January 17-31, 2024<br>January 27–February 10, 2024	0.28 (48)<br>0.23 (36)<br>0.24 (48)<br>0.24 (48)	0.25	0.02	8.24
LC_{50}	December 5–19, 2023<br>January 1–15, 2024<br>January 17-31, 2024<br>January 27–February 10, 2024	0.22 (48)<br>0.18 (48)<br>0.17 (34)<br>0.21 (48)	0.20	0.02	11.90

Table S2: Individual larval area

Bioassay date + n per treatment	Individual larval area CONTROL		Individual larval area LC_{30}		Individual larval area LC_{50}	
December 5–19, 2023	0.023	0.013	0.014	0.007	0.014	0.007
	0.017	0.010	0.007	0.010	0.006	0.007
n CONTROL = 48	0.011	0.018	0.008	0.010	0.010	0.008
n LC30 = 48	0.010	0.012	0.010	0.012	0.011	0.007
n LC50 = 48	0.014	0.015	0.008	0.006	0.008	0.008
	0.010	0.023	0.008	0.009	0.008	0.007
	0.008	0.008	0.012	0.018	0.010	0.009
	0.010	0.011	0.007	0.013	0.007	0.008
	0.013	0.014	0.013	0.011	0.008	0.010
	0.006	0.016	0.007	0.012	0.011	0.007
	0.010	0.009	0.008	0.009	0.011	0.005
	0.020	0.020	0.012	0.009	0.012	0.008

 https://doi.org/10.3390/insects17020213

	0.007	0.022	0.009	0.014	0.014	0.011
	0.008	0.011	0.011	0.009	0.012	0.017
	0.013	0.016	0.013	0.015	0.008	0.005
	0.010	0.018	0.009	0.008	0.007	0.008
	0.012	0.022	0.011	0.008	0.008	0.009
	0.014	0.016	0.008	0.009	0.006	0.008
	0.018	0.014	0.010	0.007	0.012	0.007
	0.009	0.018	0.008	0.007	0.009	0.010
	0.013	0.013	0.008	0.006	0.011	0.006
	0.013	0.009	0.011	0.009	0.009	0.010
	0.012	0.014	0.013	0.005	0.017	0.013
	0.017	0.014	0.009	0.006	0.013	0.006
January 1–15, 2024	0.017	0.009	0.009	0.008	0.010	0.007
	0.011	0.011	0.013	0.011	0.011	0.006
n CONTROL = 48	0.009	0.006	0.013	0.010	0.008	0.005
n LC30 = 36	0.008	0.010	0.004	0.006	0.010	0.005
n LC50 = 48	0.007	0.008	0.010	0.011	0.014	0.006
	0.018	0.007	0.009	0.011	0.009	0.006
	0.014	0.012	0.010	0.010	0.006	0.005
	0.023	0.007	0.007	0.006	0.008	0.006
	0.014	0.008	0.011	0.006	0.011	0.006
	0.018	0.009	0.006	0.005	0.013	0.005
	0.008	0.005	0.016	0.011	0.010	0.006
	0.012	0.010	0.006	0.006	0.009	0.005
	0.013	0.013	0.006	0.010	0.008	0.007
	0.010	0.008	0.015	0.006	0.009	0.003
	0.007	0.011	0.011	0.010	0.004	0.005
	0.009	0.010	0.008	0.008	0.009	0.005
	0.012	0.013	0.012	0.007	0.011	0.005
	0.013	0.009	0.007	0.007	0.008	0.007
	0.007	0.008			0.008	0.006
	0.017	0.008			0.005	0.005
	0.008	0.012			0.011	0.004
	0.010	0.010			0.006	0.006
	0.011	0.014			0.006	0.003
	0.010	0.012			0.008	0.004
January 17-31, 2024	0.015	0.011	0.007	0.007	0.009	0.005
	0.010	0.014	0.01	0.008	0.006	0.004
n CONTROL = 48	0.016	0.016	0.011	0.011	0.005	0.006
n LC30 = 48	0.018	0.009	0.011	0.014	0.006	0.012
n LC50 = 34	0.012	0.011	0.003	0.013	0.006	0.006
	0.007	0.008	0.006	0.005	0.007	0.006
	0.012	0.013	0.006	0.004	0.007	0.005

https://doi.org/10.3390/insects17020213

	0.005	0.005	0.018	0.011	0.016	0.009
	0.012	0.016	0.009	0.01	0.009	0.006
	0.018	0.018	0.01	0.005	0.009	0.004
	0.012	0.015	0.007	0.006	0.007	0.009
	0.007	0.017	0.006	0.005	0.006	0.011
	0.011	0.013	0.007	0.013	0.005	0.004
	0.012	0.008	0.004	0.013	0.008	0.007
	0.011	0.012	0.011	0.007	0.007	0.005
	0.009	0.012	0.008	0.009	0.007	0.008
	0.010	0.008	0.009	0.01	0.009	0.004
	0.009	0.010	0.009	0.011		
	0.017	0.008	0.007	0.009		
	0.025	0.011	0.013	0.008		
	0.009	0.013	0.014	0.007		
	0.013	0.015	0.008	0.01		
	0.011	0.006	0.01	0.011		
	0.006	0.005	0.004	0.003		
January 27–February 10, 2024<br><br>n CONTROL = 48<br>n LC30 = 48<br>n LC50 = 48	0.023	0.013	0.013	0.013	0.008	0.005
	0.012	0.011	0.012	0.005	0.010	0.007
	0.013	0.019	0.008	0.012	0.006	0.008
	0.009	0.020	0.013	0.012	0.006	0.014
	0.009	0.017	0.009	0.009	0.004	0.010
	0.014	0.012	0.011	0.006	0.006	0.011
	0.014	0.014	0.012	0.012	0.008	0.007
	0.007	0.013	0.017	0.006	0.012	0.006
	0.017	0.016	0.009	0.011	0.010	0.009
	0.015	0.011	0.019	0.010	0.008	0.007
	0.017	0.011	0.012	0.013	0.018	0.005
	0.013	0.011	0.011	0.015	0.007	0.011
	0.019	0.022	0.014	0.008	0.006	0.010
	0.012	0.022	0.006	0.014	0.010	0.013
	0.010	0.014	0.004	0.013	0.012	0.007
	0.018	0.013	0.010	0.014	0.011	0.009
	0.010	0.012	0.011	0.010	0.013	0.007
	0.021	0.013	0.005	0.007	0.012	0.014
	0.013	0.015	0.012	0.009	0.013	0.010
	0.014	0.016	0.009	0.008	0.010	0.007
	0.013	0.015	0.014	0.009	0.013	0.012
	0.009	0.012	0.015	0.008	0.011	0.011
	0.022	0.014	0.007	0.010	0.004	0.008
	0.017	0.025	0.011	0.010	0.019	0.009

https://doi.org/10.3390/insects17020213

Table S3: Individual larval and pupal stage duration

Treatment	Bioassay date	Days to pupation from sublethal treatments		Days to pupation from egg hatching		Pupal stage duration	
CONTROL	December 5–19, 2023 n=2-2-2	71	96	89	114	5	7
	January 1–15, 2024 n=1-1-1	87		105		5	
	January 17-31, 2024 n=39-39-39	61	61	79	79	5	7
		54	75	72	93	7	3
		78	54	96	72	6	7
		61	78	79	96	5	6
		102	61	120	79	5	5
		84	54	102	72	7	7
		86	78	104	96	8	6
		84	96	102	114	7	6
		107	94	125	112	4	6
		84	78	102	96	7	6
		78	75	96	93	6	7
		94	78	112	96	6	6
		61	89	79	107	7	7
		96	75	114	93	8	3
		78	54	96	72	6	7
		63	61	81	79	7	7
		84	94	102	112	7	8
		61	54	79	72	7	7
		78	98	96	116	8	7
			61		79		7
	January 27–February 10, 2024 n=64-64-53	51	51	69	69	2	7
		74	88	92	106	7	nd
		58	51	76	69	7	7
		74	53	92	71	7	nd
		51	44	69	62	5	2
		74	56	92	74	7	nd
		58	53	76	71	7	nd
		51	44	69	62	nd	6
		51	51	69	69	5	1
		51	44	69	62	7	6
		51	53	69	71	7	nd

		44	104	62	122	7	4
		51	68	69	86	7	6
		65	53	83	71	7	nd
		68	68	86	86	8	4
		51	68	69	86	1	6
		51	68	69	86	7	6
		53	113	71	131	nd	3
		68	81	86	99	6	7
		51	53	69	71	2	nd
		51	51	69	69	7	1
		58	76	76	94	7	8
		53	51	71	69	nd	2
		104	51	122	69	9	5
		68	68	86	86	6	6
		90	51	108	69	7	7
		51	51	69	69	7	7
		74	51	92	69	7	1
		65	62	83	80	7	3
		53	51	71	69	nd	2
		56	51	74	69	7	7
		62	51	80	69	6	1
LC_{30}	December 5–19, 2023 n=0-0-0	NO		NO		NO	
	January 1–15, 2024 n=2-2-2	95	85	113	103	6	7
	January 17-31, 2024 n=21-21-21	98	61	116	79	9	5
		78	114	96	132	6	4
		86	96	104	114	8	6
		54	133	72	151	7	7
		86	78	104	96	8	6
		91	94	109	112	7	6
		98	78	116	96	7	6
		78	107	96	125	6	7
		118	98	136	116	7	6
		107	133	125	151	7	7
			140		158		7
	January 27–February 10, 2024 n=48-48-42	51	68	69	86	7	4
		68	62	86	80	6	3
		81	53	99	71	7	nd
		53	58	71	76	nd	7
		74	74	92	92	7	7

https://doi.org/10.3390/insects17020213

		62	51	80	69	3	7
		51	74	69	92	7	5
		51	62	69	80	7	6
		62	62	80	80	3	6
		62	53	80	71	6	dead
		51	79	69	97	7	7
		81	53	99	71	7	nd
		58	56	76	74	7	nd
		58	58	76	76	7	7
		56	58	74	76	nd	7
		62	68	80	86	3	6
		74	51	92	69	7	7
		65	68	83	86	9	6
		62	51	80	69	3	7
		68	97	86	115	6	7
		62	68	80	86	3	6
		68	74	86	92	4	7
		68	58	86	76	6	7
		65	51	83	69	7	1
LC_{50}	December 5–19, 2023 n=0-0-0	NO		NO		NO	
	January 1–15, 2024 n=0-0-0	NO		NO		NO	
	January 17-31, 2024 n=5-5-5	133	107	151	125	7	7
		78	98	96	116	8	9
			107		125		7
	January 27–February 10, 2024 n=23-23-21	51	84	69	102	5	6
		74	51	92	69	7	7
		58	51	76	69	7	2
		51	51	69	69	7	7
		62	51	80	69	6	7
		81	58	99	76	7	7
		68	53	86	71	6	nd
		68	53	86	71	6	5
		68	53	86	71	6	nd
		81	51	99	69	7	7
		62	51	80	69	6	7
			65		83		7

Table S4: Pupae and adults area and weight

Treatment	Bioassay date	Gender	Pupal area (mm²)	Pupal weight (mg)	Adult area (mm²)	Adult weight (mg)
CONTROL	January 1–15, 2024 n=0-0-0-1-1	nd	nd	nd	8.70	8.42
	January 17-31, 2024 n=24-34-34-33-33	nd	11.10	13.69	11.90	12.70
		M	5.90	6.97	6.50	6.36
		nd	9.60	8.79	9.60	8.08
		M	7.40	7.04	7.30	6.22
		M	10.20	11.80	9.30	9.77
		F	9.70	11.72	11.40	10.16
		M	8.70	9.57	9.30	8.56
		M	9.30	9.55	9.90	8.81
		M	8.40	8.37	8.90	7.59
		M	8.20	9.53	8.40	8.50
		F	13.50	15.20	13.20	12.68
		nd	13.50	14.49	11.90	10.63
		M	7.50	7.68	7.00	6.66
		F	10.20	12.62	10.40	11.42
		nd	9.20	8.86	10.70	10.80
		F	10.40	11.72	9.50	8.61
		nd	9.40	9.51	8.80	8.07
		F	7.60	9.09	10.00	8.87
		nd	9.90	9.81	12.90	13.10
		F	11.20	14.63	8.30	7.99
		F	7.20	8.76	10.80	8.97
		nd	9.90	9.69	8.10	7.60
		F	8.40	8.18	8.00	6.58
		M	7.30	7.55	8.80	6.98
		M	8.10	7.86	7.80	7.51
		F	7.90	8.43	11.00	10.32
		F	11.60	11.50	6.60	6.44
		M	6.20	7.63	10.60	10.22
		F	9.90	11.72	10.40	10.75
		F	10.80	11.65	10.90	9.48
		nd	10.40	10.13	8.30	6.08
		M	7.70	7.00	11.40	10.25
		nd	10.20	11.64	10.00	9.09
		nd	10.40	10.50	nd	nd

https://doi.org/10.3390/insects17020213

	January 27–	nd	9.20	9.63	9.90	8.74
	February 10,	M	9.90	12.23	10.50	9.87
	2024	nd	10.90	11.03	10.20	9.84
	n=21-53-53-	F	10.40	12.21	10.50	10.83
	46-46	nd	11.10	10.95	11.50	9.60
		F	10.70	13.27	12.10	11.92
		nd	10.50	12.14	10.80	10.99
		nd	11.90	13.54	11.30	11.51
		nd	10.60	11.74	11.90	10.31
		nd	11.10	11.77	10.20	10.14
		nd	14.80	19.39	15.10	16.75
		nd	14.00	14.86	12.90	13.00
		M	12.00	15.47	11.80	12.91
		F	8.80	10.18	9.50	8.78
		nd	12.20	11.31	12.10	11.71
		nd	11.50	14.12	11.20	10.02
		M	9.40	10.71	10.40	10.85
		nd	10.00	11.63	10.60	10.19
		nd	11.80	12.17	11.50	13.55
		nd	14.40	16.55	12.10	11.77
		nd	12.50	13.44	8.30	7.46
		M	7.40	7.25	12.10	11.99
		F	10.70	13.34	12.40	11.14
		F	8.00	8.49	10.70	11.28
		nd	12.70	14.15	8.20	6.51
		M	12.00	13.10	12.90	15.00
		M	10.90	12.56	9.20	6.97
		nd	11.50	11.84	11.80	12.00
		nd	10.70	11.52	13.70	12.51
		M	8.10	7.16	10.50	11.00
		nd	14.90	17.59	15.40	14.90
		F	9.30	10.42	11.30	12.14
		nd	12.20	13.92	13.10	14.18
		nd	13.20	17.87	10.40	10.20
		nd	7.70	7.89	11.80	10.83
		nd	11.70	13.17	9.60	7.76
		nd	10.80	10.23	10.40	10.54
		M	7.00	6.85	11.70	9.75
		F	11.10	12.76	10.60	10.12
		nd	10.90	10.52	12.20	13.00
		F	11.50	16.46	14.40	16.13
		F	10.40	13.74	9.70	8.25
		F	14.80	17.83	10.60	10.42
		F	9.50	11.18	11.90	11.49

https://doi.org/10.3390/insects17020213

		nd	13.70	14.67	10.20	9.36
		M	9.20	9.43	9.90	9.51
		nd	10.70	11.27	nd	nd
		nd	10.30	12.03	nd	nd
		F	9.60	11.59	nd	nd
		nd	12.90	14.06	nd	nd
		nd	15.30	17.53	nd	nd
		nd	11.20	13.13	nd	nd
		nd	10.90	10.55	nd	nd
LC_{30}	January 1–15, 2024 n=1-2-2-2-2	F	9.60	11.03	9.90	10.31
		nd	9.80	11.07	8.60	9.59
	January 17-31, 2024 n=14-16-16-15-15	M	6.90	6.04	6.70	4.51
		M	7.20	6.29	6.80	5.62
		M	8.20	8.87	8.60	7.10
		M	9.50	9.73	9.20	8.32
		F	9.20	10.35	9.50	9.40
		nd	9.40	9.83	9.00	9.00
		F	8.80	9.77	8.90	8.86
		M	5.70	4.44	4.90	3.47
		nd	8.60	7.65	8.50	7.01
		F	9.50	9.03	9.30	8.53
		M	8.30	9.79	10.40	10.37
		F	8.90	11.21	10.90	8.90
		M	8.90	9.69	7.70	7.32
		F	7.90	8.11	8.00	6.87
		F	8.60	8.25	9.70	9.44
		F	9.40	10.94	nd	nd
	January 27–February 10, 2024 n=22-41-41-42-42	nd	nd	nd	12.60	11.62
		nd	11.20	13.83	15.10	14.45
		F	13.50	16.98	10.10	7.29
		M	8.50	8.87	10.70	12.34
		nd	13.10	14.78	9.70	9.60
		M	13.70	15.00	14.30	14.90
		nd	14.70	16.73	12.80	13.70
		nd	12.60	15.06	12.20	11.70
		M	7.80	9.00	9.90	8.20
		nd	12.30	13.55	13.00	11.60
		F	13.30	19.76	16.00	16.71
		nd	12.60	14.37	11.40	12.80
		nd	9.00	9.96	9.00	8.75
		nd	13.00	15.42	15.50	17.12
		F	15.00	14.93	13.70	12.90
		F	11.80	17.79	14.10	15.02

https://doi.org/10.3390/insects17020213

		M	9.30	9.64	10.60	11.32
		M	9.70	10.43	8.40	8.42
		M	12.00	13.81	11.40	12.19
		F	9.30	11.11	10.50	8.94
		M	8.70	10.17	11.00	11.02
		nd	10.00	9.92	9.90	10.19
		nd	9.70	10.77	10.00	8.94
		F	8.80	10.21	8.80	8.82
		nd	11.00	12.24	9.60	9.06
		M	8.60	9.62	9.10	9.08
		F	9.60	11.82	10.50	10.07
		F	12.30	13.20	8.80	8.48
		nd	12.70	14.61	10.90	10.66
		F	10.20	12.73	12.90	12.45
		nd	12.10	14.27	10.60	10.45
		nd	8.00	7.62	12.70	15.10
		nd	11.70	17.43	11.30	11.49
		nd	11.50	13.25	11.80	14.85
		F	13.30	17.00	12.80	12.62
		nd	14.00	14.60	13.10	13.75
		F	12.40	15.82	10.30	10.61
		nd	11.30	11.93	7.70	5.98
		M	7.70	6.90	10.30	10.37
		M	10.60	12.72	13.40	13.33
		F	13.20	15.28	10.90	10.34
		nd	9.90	12.00	10.60	9.57
LC_{50}	January 1–15, 2024 n=0-0-0	NO	NO	NO	NO	NO
	January 17-31, 2024 n=4-4-4-4-4	M	6.60	7.54	7.40	6.59
		M	7.60	7.91	6.90	6.92
		M	8.70	8.55	8.20	7.17
		M	8.30	8.46	9.30	7.14
	January 27–February 10, 2024 n=9-22-22-19-19	nd	9.10	9.33	9.30	8.13
		M	8.20	8.50	8.80	7.52
		nd	11.10	14.37	12.30	12.43
		nd	12.60	14.44	13.50	12.52
		F	9.70	9.63	7.80	9.00
		F	9.20	9.27	8.90	8.34
		F	12.40	13.68	11.30	12.41
		M	12.70	12.29	10.30	10.65
		M	9.00	9.65	8.90	5.67
		F	11.90	13.05	11.30	11.25
		M	12.50	14.09	12.70	12.21

		nd	12.60	14.35	7.40	6.50
		nd	8.00	7.16	14.60	14.31
		nd	14.80	17.22	11.10	11.95
		nd	12.50	13.20	10.70	12.68
		nd	12.20	14.78	9.50	9.83
		nd	13.00	15.23	14.90	15.77
		nd	10.20	11.12	14.90	15.22
		nd	11.90	13.88	12.40	13.82
		nd	14.60	18.46	nd	nd
		nd	15.30	17.18	nd	nd
		F	13.50	16.10	nd	nd

Table S5: Global survival analysis

**Treatment**	**Bioassay date**	**Survival (days)**		**Censorship code**	
CONTROL	December 5–19, 2023 n=48	33	14	1	1
		19	26	1	1
		140	5	1	1
		21	21	1	1
		21	1	1	1
		23	1	1	1
		19	28	1	1
		1	1	1	1
		40	28	1	1
		40	168	1	1
		19	1	1	1
		28	35	1	1
		30	1	1	1
		37	28	1	1
		19	35	1	1
		35	16	1	1
		26	14	1	1
		19	14	1	1
		33	1	1	1
		37	37	1	1
		19	19	1	1
		28	19	1	1
		21	16	1	1
		14	9	1	1

https://doi.org/10.3390/insects17020213

	January 1–15, 2024 n=13	10	13	1	1
		10	10	1	1
		6	24	1	1
		13	154	1	1
		20	10	1	1
		13	13	1	1
			13		1
	January 17-31, 2024 n=48	165	122	1	1
		165	165	1	1
		122	122	1	1
		165	8	1	1
		1	97	1	0
		122	165	1	1
		122	165	1	1
		125	165	1	1
		6	165	1	1
		122	138	1	1
		165	165	1	1
		122	165	1	1
		1	165	1	1
		1	165	1	1
		122	165	1	1
		122	165	1	1
		165	1	1	1
		122	8	1	1
		122	174	1	1
		125	174	1	1
		122	1	1	1
		125	174	1	1
		122	380	1	1
		125	477	1	1
	January 27–February 10, 2024 n=127	3	36	1	0
		36	36	0	0
		12	43	1	0
		5	36	1	0
		36	115	0	1
		97	36	0	0
		112	115	1	1
		36	155	0	1
		122	155	1	1
		36	155	0	1
		112	155	1	1
		122	36	1	0
		36	36	0	0

https://doi.org/10.3390/insects17020213

	36	155	0	1
	112	155	1	1
	36	155	0	1
	36	155	0	1
	122	164	1	1
	79	36	0	0
	36	43	0	0
	36	115	0	0
	122	5	1	1
	122	112	1	1
	36	115	0	1
	128	78	1	0
	36	36	0	0
	5	36	1	0
	36	36	0	0
	155	3	1	1
	80	78	1	0
	112	112	1	1
	36	3	0	1
	36	3	0	1
	5	164	1	1
	112	5	1	1
	36	50	0	1
	155	36	1	0
	36	164	0	1
	155	316	1	1
	155	36	1	0
	78	50	1	1
	5	370	1	1
	3	36	1	0
	93	449	1	1
	155	36	1	0
	78	449	0	1
	155	36	1	0
	8	457	1	1
	36	457	0	1
	155	36	1	0
	78	483	0	1
	155	87	1	0
	155	492	1	1
	112	492	1	1
	112	80	1	1
	155	492	1	1
	36	36	0	0

https://doi.org/10.3390/insects17020213

		155	36	1	0
		36	36	0	0
		112	36	1	0
		155	492	1	1
		3	521	1	1
		36	36	0	0
			36		0
LC_{30}	December 5–19, 2023 n=48	23	14	1	1
		26	26	1	1
		12	12	1	1
		30	1	1	1
		5	19	1	1
		19	12	1	1
		12	35	1	1
		1	12	1	1
		5	16	1	1
		21	16	1	1
		21	12	1	1
		19	19	1	1
		14	1	1	1
		16	19	1	1
		1	33	1	1
		35	12	1	1
		21	5	1	1
		21	23	1	1
		16	23	1	1
		14	19	1	1
		28	19	1	1
		14	21	1	1
		19	1	1	1
		21	16	1	1
	January 1–15, 2024 n=37	1	3	1	1
		1	3	1	1
		1	10	1	1
		1	6	1	1
		1	6	1	1
		1	3	1	1
		1	148	1	1
		1	13	1	1
		1	6	1	1
		1	13	1	1
		1	8	1	1
		1	154	1	1
		3	13	1	1

https://doi.org/10.3390/insects17020213

		6	3	1	1
		15	3	1	1
		10	7	1	1
		3	7	1	1
		6	8	1	1
			3		1
	January 17-31, 2024 n=48	1	1	1	1
		125	1	1	1
		122	182	1	1
		122	165	1	1
		1	1	1	1
		4	4	1	1
		132	165	1	1
		6	6	1	1
		122	379	1	1
		122	6	1	1
		1	1	1	1
		1	1	1	1
		1	1	1	1
		125	8	1	1
		122	122	1	0
		132	8	1	1
		122	125	1	1
		125	152	1	1
		165	152	1	0
		11	11	1	1
		1	39	1	1
		4	4	1	1
		125	1	1	1
		152	1	1	1
	January 27–February 10, 2024 n=96	2	12	1	1
		4	154	1	1
		115	115	0	1
		4	18	1	1
		112	4	1	1
		4	4	1	1
		2	4	1	1
		112	4	1	1
		122	115	1	1
		4	115	1	1
		112	4	1	1
		122	154	1	1
		122	36	1	0
		36	2	0	1

https://doi.org/10.3390/insects17020213

		36	164	0	1
		122	164	1	1
		2	36	1	0
		36	164	0	1
		4	4	1	1
		122	384	1	1
		4	56	1	1
		112	384	1	1
		122	384	1	1
		122	36	1	0
		2	420	1	1
		122	4	1	1
		122	457	1	1
		128	4	1	1
		2	115	1	1
		28	483	1	1
		154	500	1	1
		36	77	0	0
		112	507	1	1
		112	2	1	1
		154	507	1	1
		2	2	1	1
		112	82	1	0
		4	4	1	1
		154	4	1	1
		28	4	1	1
		112	2	1	1
		4	511	1	1
		36	511	0	1
		154	4	1	1
		2	2	1	1
		4	4	1	1
		154	521	1	1
		2	4	1	1
LC_{50}	December	1	12	1	1
	5–19, 2023	1	5	1	1
	n=48	14	1	1	1
		33	21	1	1
		1	12	1	1
		23	14	1	1
		1	26	1	1
		1	5	1	1
		14	12	1	1
		1	33	1	1

	12	21	1	1
	21	9	1	1
	23	23	1	1
	12	12	1	1
	1	26	1	1
	26	28	1	1
	19	5	1	1
	1	1	1	1
	5	5	1	1
	1	1	1	1
	19	26	1	1
	12	23	1	1
	19	1	1	1
	5	28	1	1
January 1–15, 2024 n=48	3	1	1	1
	3	1	1	1
	3	1	1	1
	3	1	1	1
	6	1	1	1
	6	1	1	1
	6	1	1	1
	3	1	1	1
	10	1	1	1
	49	1	1	1
	122	1	1	1
	6	1	1	1
	10	1	1	1
	8	1	1	1
	10	1	1	1
	3	1	1	1
	8	1	1	1
	8	1	1	1
	8	1	1	1
	13	1	1	1
	8	1	1	1
	1	1	1	1
	1	1	1	1
	1	1	1	1
January 17-31, 2024 n=34	1	1	1	1
	1	4	1	1
	1	1	1	1
	4	4	1	1
	152	1	1	1
	125	1	1	1

https://doi.org/10.3390/insects17020213

	132	1	1	1
	4	22	1	1
	28	138	1	1
	11	4	1	1
	1	1	1	1
	1	138	1	1
	6	6	1	1
	6	4	1	1
	4	4	1	1
	4	4	1	1
	39	1	1	1
January 27–February 10, 2024 n=80	2	2	1	1
	28	2	1	1
	115	2	1	1
	2	4	1	1
	2	4	1	1
	4	36	1	0
	36	2	0	1
	7	2	1	1
	4	2	1	1
	2	4	1	1
	112	2	1	1
	36	66	0	1
	4	2	1	1
	2	154	1	1
	18	77	1	0
	2	154	1	1
	122	154	1	1
	4	2	1	1
	4	2	1	1
	4	36	1	0
	4	12	1	1
	122	4	1	1
	2	154	1	1
	2	154	1	1
	112	36	1	0
	12	2	1	1
	2	4	1	1
	163	115	1	1
	4	154	1	1
	4	4	1	1
	12	115	1	1
	383	115	1	1
	4	36	1	0

https://doi.org/10.3390/insects17020213

	483	7	1	1
	8	36	1	0
	36	154	0	1
	4	507	1	1
	2	36	1	0
	77	521	1	1
	154	2	1	1

Abbreviations

nd = no data

F = female

M = male

Disclaimer/Publisher's Note: The statements, opinions and data contained in all publications are solely those of the individual author(s) and contributor(s) and not of MDPI and/or the editor(s). MDPI and/or the editor(s) disclaim responsibility for any injury to people or property resulting from any ideas, methods, instructions or products referred to in the content.
